# Mental health of Italian university students: an overview of reviews

**DOI:** 10.1192/j.eurpsy.2026.11825

**Published:** 2026-07-31

**Authors:** S. Magliocca, A. Provera, M. Bani, M. Bellani, C. Buizza, C. Callegari, A. Compare, A. Brugnera, A. Delle Fave, A. Ghilardi, G. Mirabella, D. Maffeis, C. Mucci, C. Piccioni, P. Cancelli, B. Rosina, S. Simonetta, M. G. Strepparava, E. Trotti, P. Profeta, S. Testa, A. Maravita, V. Velasco, F. Madeddu, R. Calati

**Affiliations:** 1 Department of Psychology; 2 School of Medicine and Surgery, University of Milan-Bicocca , Milan; 3 Department of Medicine and Technological Innovation, University of Insubria, Varese; 4 Department of Clinical and Experimental Sciences, University of Brescia, Brescia; 5 Department of Medicine and Surgery, University of Insubria, Varese; 6 Department of Human and Social Sciences, University of Bergamo, Bergamo; 7 Dipartimento di Fisiopatologia Medico-Chirurgica e dei Trapianti, University of Milan, Milan; 8 Projects Coordination Office, Politecnico delle Arti di Bergamo, Bergamo; 9 Department of Social and Political Sciences, Bocconi University; 10 COSP – Centre for Orientation to Studies and Careers; 11 Department of Philosophy, University of Milan, Milan; 12 SSD Psicologia Clinica, Fondazione IRCCS San Gerardo dei Tintori, Monza, Italy; 13 Department of Adult Psychiatry, Nîmes University Hospital, Nîmes, France

## Abstract

**Introduction:**

University students’ mental health is a global concern, with high prevalence of anxiety, depression, and stress. In Italy, however, no comprehensive synthesis is available. We conducted an overview of reviews to summarize prevalence rates, risk and protective factors for mental health outcomes, and the characteristics and effectiveness of counseling services.

**Objectives:**

To describe the state of mental health among Italian university students, identify risk/protective factors, and synthesize evidence on counseling services and interventions.

**Methods:**

Following PRIOR guidelines, we systematically searched PubMed, Web of Science, Scopus, and PsycINFO. From 96 records, 9 reviews met inclusion criteria (6 systematic, 2 with meta-analyses, 1 narrative, 1 rapid, 1 scoping). Reviews included Italian university students (2004–2023) and reported mental health outcomes or interventions.

**Results:**

Prevalence rates were high: anxiety (11–65%), depression (9–73%), stress (40–89%), and sleep disturbances (9–65%); suicidal ideation affected ~7%. Symptoms increased during COVID-19, with poorer sleep and more internalizing and externalizing difficulties. Risk factors included female gender, younger age, first-year status, minority sexual orientation, health and family vulnerabilities, financial strain, academic pressure, and low social support. Protective factors were resilience, supportive relationships, inclusive campus climates, physical activity, and mindfulness techniques. Interventions were heterogeneous, from psychodynamic and CBT counseling to group approaches, innovative techniques, and prevention programs. Counseling services were mostly short-term, free, and diversified. Reviews reported improvements in well-being, coping, distress, and academic functioning, though standardized outcomes and long-term evidence were limited.

**Conclusions:**

Italian university students showed a high burden of mental health problems, worsened by COVID-19. Risk and protective factors spanned individual, relational, and contextual domains. Counseling services appeared beneficial but lacked standardized and long-term evaluations. Strengthening evidence-based, accessible, and sustainable interventions in universities is a public health priority.

**Disclosure of Interest:**

None Declared

